# ﻿Two new species of feather mites (Acariformes, Astigmata) from the black-tailed godwit, *Limosalimosa* (Charadriiformes, Scolopacidae), in Korea

**DOI:** 10.3897/zookeys.1088.80307

**Published:** 2022-03-08

**Authors:** Yeong-Deok Han, Sergey V. Mironov, Gi-Sik Min

**Affiliations:** 1 Department of Biological Sciences, Inha University, Incheon 22212, Republic of Korea Inha University Incheon Republic of Korea; 2 Research Center for Endangered Species, National Institute of Ecology, Gowol-gil 23, Yeongyang-gun, 36531, Republic of Korea National Institute of Ecology Yeongyang-gun Republic of Korea; 3 Zoological Institute, Russian Academy of Sciences, Universitetskaya Embankment 1, Saint-Petersburg, 199034, Russia Zoological Institute, Russian Academy of Sciences Saint Petersburg Russia

**Keywords:** *Alloptes*, COI, feather mite, Korea, *Phyllochaeta*, systematics

## Abstract

Two new species of feather mites are described from two individuals of the black-tailed godwit, *Limosalimosa* (Linnaeus, 1758), in Korea: Alloptes (Conuralloptes) neolimosae**sp. nov.** (Analgoidea, Alloptidae) and *Phyllochaetalimosae***sp. nov.** (Pterolichoidea, Syringobiidae). Males of A. (C.) neolimosae**sp. nov.** are distinguished from A. (C.) limosae in having the hysteronotal shield with a straight anterior margin, setae *h2* enlarged and slightly flattened in the basal half, and the terminal lamella monotonously transparent without sclerotized patches; females differ in having legs IV with ambulacral discs extending to or slightly beyond the level of setae *f2*. The discovery of *P.limosae***sp. nov.** represents the first record of the feather mite genus *Phyllochaeta* on godwits of the genus *Limosa* Brisson, 1760 (Scolopacidae, Limosinae). Males of *P.limosae***sp. nov.** are distinguished from *P.secunda* in having the terminal cleft semi-ovoid with a length-to-width ratio of 1.7, and the terminal membranes with 15 or 16 finger-shaped denticles; females differ in having the hysteronotal shield bearing faint longitudinal striations in the posterior third and lacking lacunae, and setae *c1* situated posterior to the level of setae *c2*. Additionally, we obtained partial sequences of the mitochondrial cytochrome *c* oxidase subunit I (COI) gene from A. (C.) neolimosae**sp. nov.** and estimated genetic distances from 10 other *Alloptes* species based on comparisons of COI sequences.

## ﻿Introduction

Feather mites comprise two superfamilies (Analgoidea and Pterolichoidea) of astigmatan mites within the order Sarcoptiformes and are permanent parasites or commensal ectosymbionts that colonize particular microhabitats in the plumage and on the skin of birds (Gaud and Atyeo 1996; Dabert and Mironov 1999; Proctor 2003). Species and supraspecific taxa of feather mites generally exhibit a high level of host-specificity that is apparently caused by specialization to particular microhabitats and dispersal primarily by direct physical contact between host individuals (Mironov and Dabert 1999; Proctor and Owens 2000; Dabert 2005).

The black-tailed godwit, *Limosalimosa* (Linnaeus, 1758) is a wader that is widely distributed in the Palearctic realm but has a disjunctive breeding range (Engelmoer and Roselaar 1998; Gill et al. 2007). This bird is subdivided into four subspecies [*L.lbohaii* Zhu, Piersma, Verkuil & Conklin, 2020, *L.L.islandica* Brehm, 1931, *L.L.limosa* (Linnaeus, 1758), and *L.L.melanuroides* Gould, 1846] based on morphological and genetic characteristics, and is also recognized by classification authority such as the International Ornithological Congress (IOC) (Gill et al. 2021; Zhu et al. 2021). In Korea, this bird species occurs as a passage migrant generally observed during autumn and spring migrations (Lee et al. 2014).

Records of various feather mites associated with *L.limosa* are known in Europe, Africa, and northern Asia. To date, 12 feather mite species have been reported from *L.limosa*, among which seven are specific to this species or to the genus *Limosa* Brisson, 1760 (Bedford 1936; Dubinin 1951, 1956; Gaud 1958, 1972, 1973; Gaud and Till 1961; Gaud and Mouchet 1963; Vasyukova and Mironov 1990, 1991; Dabert and Ehrnsberger 1999; Dabert 2003). In Korea, four feather mite species have been recorded from this bird species (Han and Min 2019a, 2019b): Alloptes (Conuralloptes) limosae Dubinin, 1951, *Avenzoariapunctata* Gaud, 1972, *Bregetovialimosae* (Buchholz, 1869), and *Montchadskianabuchholzi* (Canestrini, 1878).

In this paper, we describe two new species of the genera *Alloptes* Canestrini, 1879 and *Phyllochaeta* Dubinin, 1951, which were found on two individuals of *L.limosae* in Korea. Additionally, we present DNA barcodes for the mitochondrial cytochrome *c* oxidase subunit I (COI) gene sequences from the newly described *Alloptes* species and estimate genetic distances with other *Alloptes* species based on comparison of COI sequences.

## ﻿Materials and methods

### ﻿Material sampling

Carcasses of two black-tailed godwits (CNWARC no. CN12-402, and CN17-265) were provided by the Chungnam Wild Animal Rescue Center (**CNWARC**). These birds were initially rescued in Asan and Seosan-si (si = City), Chungcheongbuk-do (do = Province) but later died during the course of treatment. Mite samples were collected from wing feathers of the two godwits under a dissecting microscope using a preparation needle and thereafter preserved in 95% ethanol. These were subsequently cleared in 10% lactic acid at room temperature for one day and then mounted on microscope slides using PVA mounting medium (BioQuip, Rancho Dominguez, California, USA). Mite specimens were observed under a light microscope (DM2500; Leica, Wetzlar, Germany). Figures were drawn and photographed with a drawing tube and microscopic digital camera (7D; Canon, Tokyo, Japan) attached to a light microscope, respectively, and were assembled and edited using Adobe Illustrator and Photoshop CS5 (Adobe Systems Incorporated, San Jose, California, USA).

Descriptions of two new species are presented herein following the standard formats adopted for the families Alloptidae and Syringobiidae (Dabert 2003; Mironov and Palma 2006; Hernandes et al. 2017; Han et al. 2021). Terminology, idiosomal, and leg chaetotaxy follow Gaud and Atyeo (1996), with minor corrections for the coxal setae proposed by Norton (1998). All measurements are in micrometers (μm). All examined specimens are deposited at the National Institute of Biological Resources (NIBR), Korea. The classification and scientific names of birds follow Gill et al. (2021).

### ﻿DNA sequencing and molecular analysis

Genomic DNA of the new *Alloptes* species was extracted from the whole body of two isolated individuals found on CNWARC no. CN12-402, and a single leg per individual from two individuals discovered on CNWARC no. CN17-265, using a Tissue DNA Purification Kit (Cosmogenetech Inc., Seoul, Korea) according to the manufacturer’s instructions (Table 1). The exoskeletons remaining after DNA extraction were mounted on microscope slides using methods described above for species identification.

**Table 1.** List of *Alloptes* species used in the molecular analysis and respecitve references.

Species	Collection host	Collection locaity	GenBank accesession No.	Reference
Alloptes (Alloptes) aschizurus	*Chionisalbus*	King George Island, Antarctica	MZ489638	Han et al. 2021
Alloptes (Apodalloptes) orthogramme	*Actitishypoleucos*	Cheongyang-gun, Korea	MK456598	Han and Min 2019a
Alloptes (Conuralloptes) calidridis	*Calidrisalpina*	Michigan, USA	KU203101	Klimov et al. 2017
Alloptes (C.) chionis	*Chionisalbus*	King George Island, Antarctica	MZ489639	Han et al. 2021
Alloptes (C.) limosae	*Limosalimosa*	Asan-si, Korea	MK456600	Han and Min 2019a
Alloptes (C.) neolimosae sp. nov.	*Limosalimosa*	Asan-si, Korea	OM102971–OM102972	Present study
		Seosan-si, Korea	OM102973–OM102974	
Alloptes (C.) procerus	*Numeniusphaeopus*	Taean-gun, Korea	MK456602	Han and Min 2019a
Alloptes (Sternalloptes) antarcticus	*Stercorariusmaccormicki*	King George Island, Antarctica	MZ489641	Han et al. 2021
Alloptes (S.) fauri	*Laruscrassirostris*	Ulleung-gun, Korea	MK456605	Han and Min 2019a
Alloptes (S.) obtusolobus	*Larusvegae*	Irkutskaya Oblast, Russia	KU203100	Klimov et al. 2017
Alloptes (S.) stercorarii	*Stercorariusparasiticus*	Kongsfjorden, Svalbard	KF018833	Dabert et al. 2015

A COI barcode fragment was amplified using KOD-Plus (Toyobo, Osaka, Japan) in conjunction with two universal primers (bcd05F [5´-TTTTCTACHAAYCATAAAGATATTGC-3´] and bcd04R [5´-TATAAACYTCDGGATGNCCAAAAAA-3´]) under the following conditions: an initial denaturation for 2 min at 94 °C; 40 cycles at 98 °C for 15 s, 50 °C for 30 s, and 68 °C for 60 s; and a final extension at 68 °C for 5 min (Dabert et al. 2008). The amplified products were sequenced using an ABI3100 automated sequencer (Perkin Elmer, Foster City, California, USA). Sequence assembly, alignment, and trimming were performed using Geneious v. 8.1.9 software (Biomatters, Auckland, New Zealand) (Kearse et al. 2012). However, despite performing similar procedures for the newly described *Phyllochaeta* species, we were unable to obtain the corresponding COI sequences.

COI sequences obtained for the new *Alloptes* species were aligned with those of 10 other *Alloptes* species registered in the National Center for Biotechnology Information (NCBI) database using Geneious v. 8.1.9 (Table 1). Pairwise distances between sequences were computed using a Kimura two-parameter (K2P) substitution model with Mega X v. 10.1.7 software (Kumar et al. 2018).

## ﻿Systematic account

### ﻿Superfamily Analgoidea Trouessart & Mégnin, 1884

**Family Alloptidae Gaud, 1957**

**Genus *Alloptes* Canestrini, 1879**

#### Subgenus Conuralloptes Gaud, 1972

##### ﻿ Alloptes (Conuralloptes) neolimosae sp. nov.

Taxon classificationAnimaliaSarcoptiformesAlloptidae

6ED6591D-422E-5F5D-B9EF-CB734F200285

http://zoobank.org/7C4BDC0F-CC75-4FC0-B6CF-FA42D6BA7FA3

###### Type material.

**Male *holotype*** (NIBR no. NIBRIV0000895968), 2 male and 3 female paratypes (NIBR no. NIBRIV0000895969–NIBRIV0000895973) from flight feathers on wings of *Limosalimosa* (Charadriiformes, Scolopacidae), Korea, Chungcheongnam-do, Asan-si, 36°48'58"N, 127°2'45"E, 18 May 2017, collected by Han Y.-D.; 3 male and 3 female paratypes (NIBR no. NIBRIV0000895978–NIBRIV0000895983) from the same host species, Korea, Chungcheongnam-do, Seosan-si, 37°0'12"N, 126°24'5"E, 6 July 2012, collected by Han Y.-D.

###### Description.

**Figure 1.**
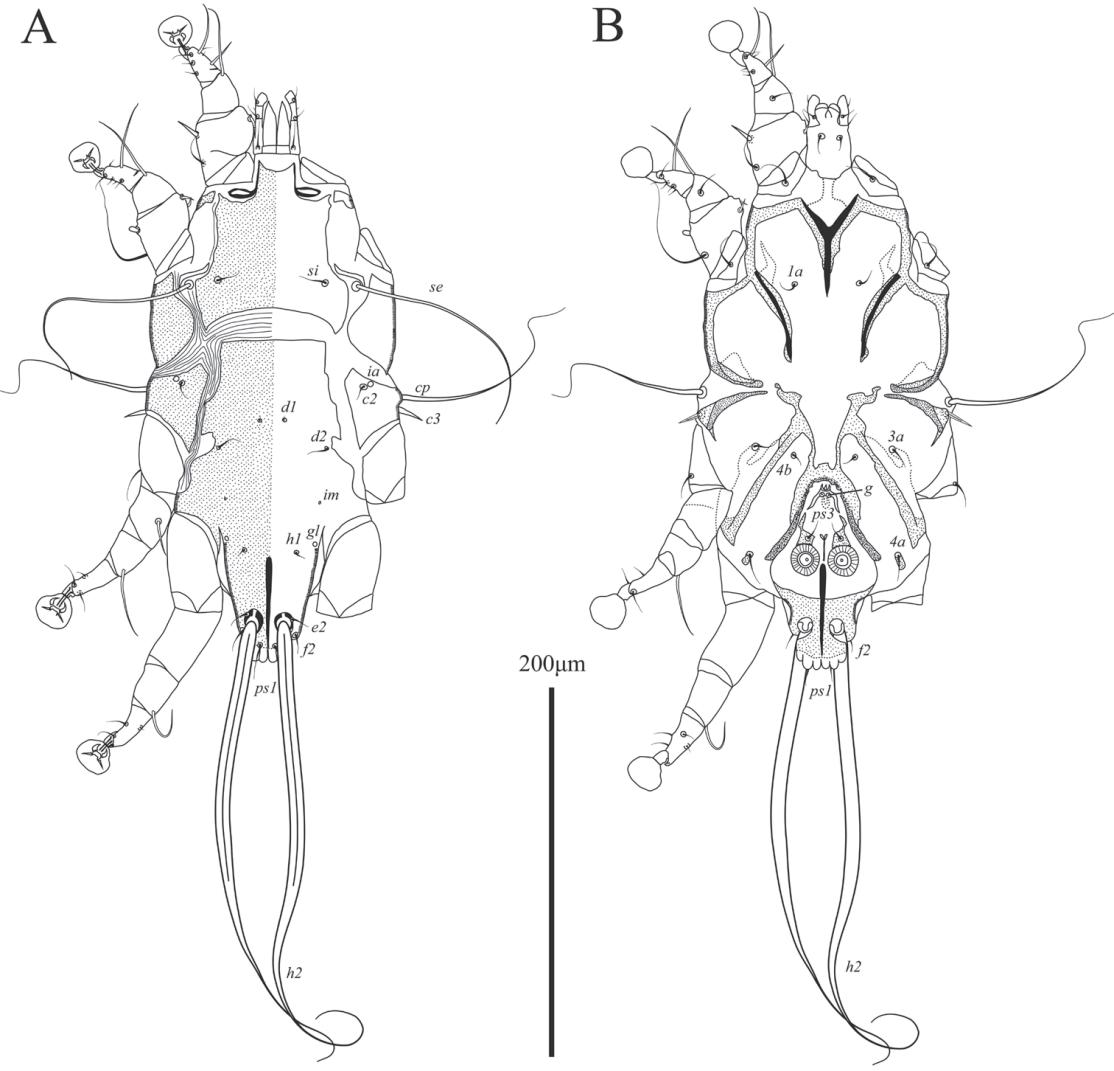
Alloptes (Conuralloptes) neolimosae sp. nov., male **A** dorsal view **B** ventral view.

**Male** (Figs 1, 3A–E, 4A, B, D; holotype, range for 4 paratypes in parentheses). Idiosoma, length × width, 283 (270–305) × 143 (135–153). Length of hysterosoma 163 (158–168). Prodorsal shield: length 83 (81–90), width at posterior margin 85 (80–89), posterior margin concave, distance between setae *se* 98 (96–104). Hysteronotal shield (Fig. 1A): greatest length 173 (163–185), width at anterior margin 60 (57–62), anterior margin straight, surface without ornamentation, lateral margins with small incision at bases of setae *d2*. Distance between prodorsal and hysteronotal shields along midline 19 (10–20). Subhumeral setae *c3* narrowly lanceolate, 16 (13–16) × 2 (2–2.5). Posterior part of opisthosoma gradually attenuate posteriorly, without terminal enlargement, width of distal part at level of setae *h2* 37 (35–39). Lateral borders of opisthosoma at level of articulation between trochanter and femur IV S-shaped. Interlobar septum 47 (46–49) in length. Terminal lamella monotonously transparent, with six festoons, incision between inner pair slit-like. Setae *h3* absent, setae *ps2* greatly reduced (Fig. 3A). Setae *h2* slightly enlarged and flattened in basal half, greatest width 5 (5.5–6.5) (Figs 1A, 4D). Dorsal measurements: *c2*:*d2* 37 (41–43), *d2*:*ps1* 128 (118–128).

Epimerites I fused into a Y (Fig. 1B). Pregenital sclerites connected to inner ends of epimerites IIIa and anterior margin of paragenital arch, distant from each other (Fig. 3A). Genital-anal field 96 (91–103) in length. Genital arch 17 (17–20) × 15 (14–19). Coxal setae *4b* situated posterior to setae *3a*. Setae *4a* on small roughly ovate sclerites. Ventral measurements: *3a*:*4b* 5 (3–5), *4b*:*g* 20 (20–26), *4b*:*4a* 56 (55–63), *g*:*ps3* 26 (23–27), *ps3*:*ps1* 61 (59–66), *4a*:*4a* 88 (85–91).

Setae *mG*I and *mG*II spine-like, with acute and bluntly rounded apices, respectively (Fig. 3B, C). Leg IV 138 (138–150) in length. Tarsus IV 27 (28–31) in length, with claw-like apical process; setae *d* and *e* minute spine-like, seta *e* situated near base of apical process (Fig. 3E).

**Figure 2.**
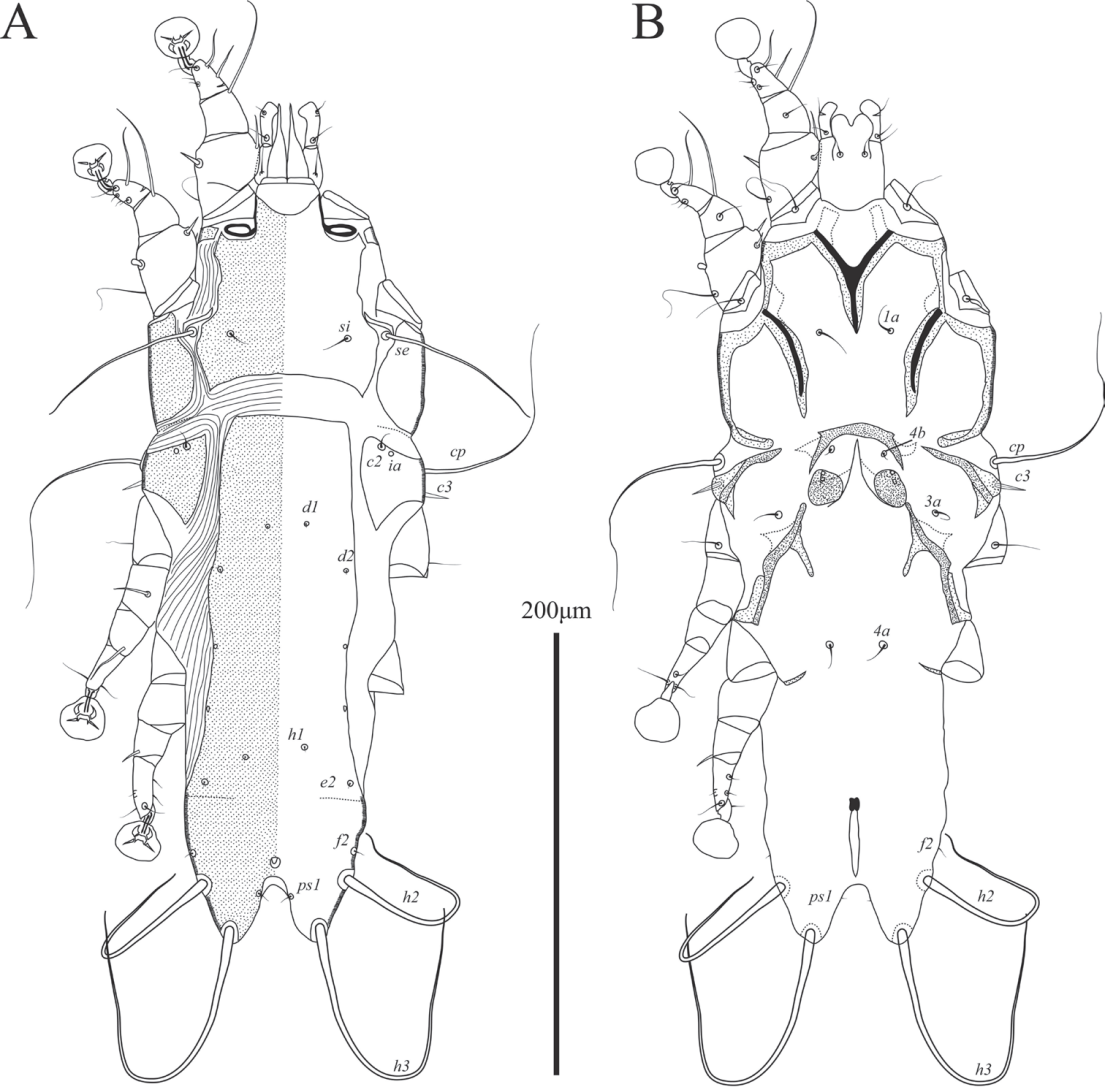
Alloptes (Conuralloptes) neolimosae sp. nov., female **A** dorsal view **B** ventral view.

**Figure 3.**
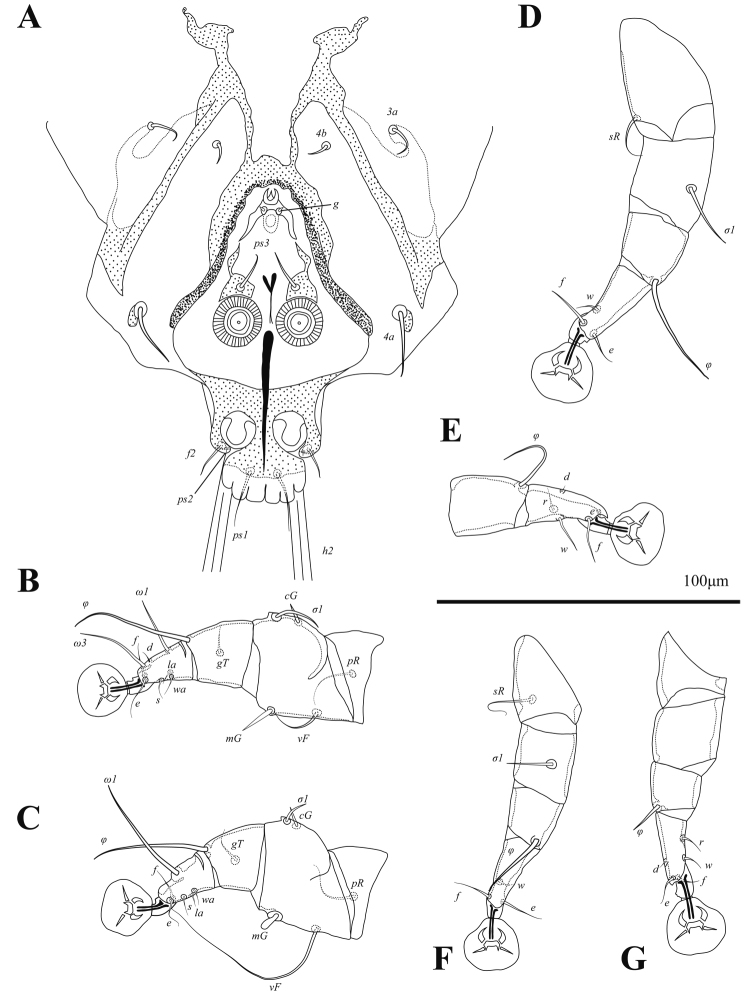
Alloptes (Conuralloptes) neolimosae sp. nov., details **A** opisthosoma of male, dorsal view **B** leg I of male **C** leg II of male **D** leg III of male **E** tibia and tarsus IV of male **F** leg III of female **G** leg IV of female.

**Figure 4.**
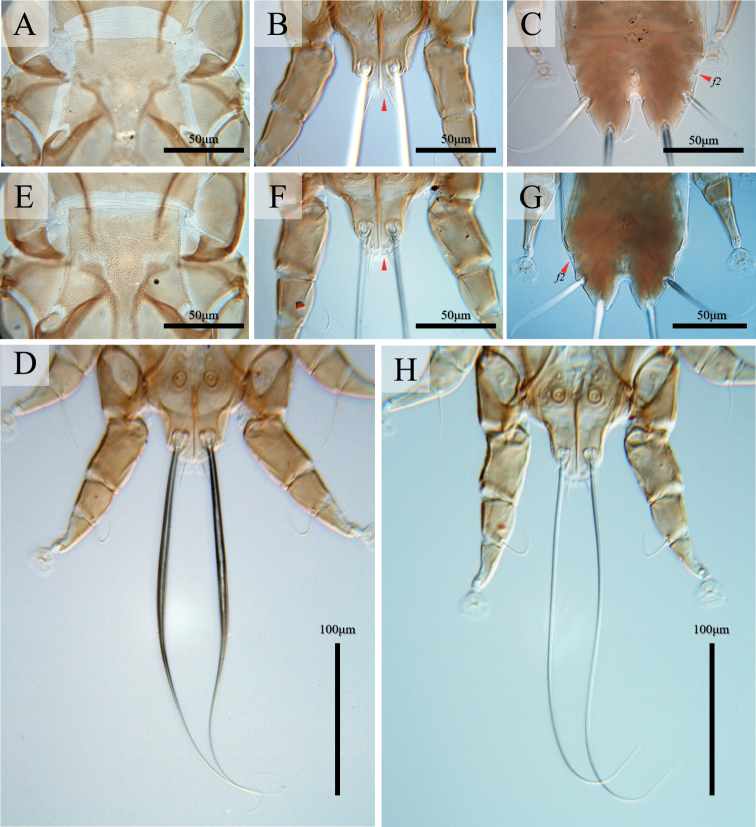
*Alloptes* species **A–D**Alloptes (Conuralloptes) neolimosae sp. nov. **E–H**A. (C.) limosae. **A, E** hysteronotal shield of males **B, F** opisthosomal lobes of males **C, G** opisthosomal lobes of females **D, H** setae *h2* of males.

**Female** (Figs 2, 3F–G, 4C; range for 5 paratypes). Idiosoma, length × width, 340–360 × 130–145 (Fig. 2A). Hysterosoma 230–245 long. Prodorsal shield: shaped as in male, 80–85 × 78–90, distance between setae *se* 91–100. Setae *c3* lanceolate, 12–14 × 2–2.5. Hysteronotal shield: 240–255 × 54–57, anterior margin straight or slightly concave, surface without ornamentation. Setae *h1* situated anterior to setae *e2*. Setae *f2* and *ps1* present. Distance between prodorsal and hysteronotal shields along midline 17–24. Supranal concavity ovate, separated from terminal cleft. Opisthosomal lobes well developed, approximately as long as wide at base, terminal cleft as an inverted U, 27–30 long (Fig. 2A). Dorsal measurements: *c2*:*d2* 55–61, *d2*:*e2* 98–103, *e2*:*h2* 43–50, *h2*:*h3* 20–23, *h2*:*h2* 62–70, *h3*:*h3* 35–45.

Bases of trochanters I, II flanked by narrow sclerotized bands connecting bases of corresponding epimerites (Fig. 2B). Epimerites IVa barely distinct. Epigynum bow-shaped, 25–26 × 45–47. Ambulacral discs of legs IV extending to or slightly beyond level of setae *f2* (Figs 2B, 4C).

###### Differential diagnosis.

Among the 23 previously described species in the subgenus Conuralloptes (Gaud 1972; Vasyukova and Mironov 1991; Mironov and Palma 2006; Han et al. 2021), the new species Alloptes (C.) neolimosae sp. nov. is most similar to A. (C.) limosae Dubinin, 1951 found on the same host, *L.limosa*. These *Alloptes* species share the following characteristics in males: the pregenital sclerites connecting the paragenital arch and inner ends of epimerites IIIa are widely separated from each other, and setae *ps3* are situated anterior to coxal setae *4a* (Dubinin 1951; Gaud 1972; Han and Min 2019a). Alloptes (C.) neolimosae sp. nov. differs from A. (C.) limosae in having the following characteristics: in males, the anterior margin of the hysteronotal shield is straight, the lateral margins of the opisthosoma are S-shaped, setae *h2* are enlarged (5.5–6.5 wide) and slightly flattened in the basal half, and the terminal lamella is monotonously transparent without sclerotized patches (Fig. 4A, B, D); in females, the terminal cleft is longer (27–30 long), and ambulacral discs of legs IV extend to or slightly beyond the level of setae *f2* (Fig. 4C). In males of A. (C.) limosae, the anterior margin of the hysteronotal shield is slightly concave, the lateral borders of the opisthosoma are straight or slightly concave, setae *h2* are rod-shaped without noticeable expansion, and the terminal lamella has three pairs of small crescent-shaped sclerites (Fig. 4E, F, H); in females, the terminal cleft is shorter (20–21 long), and ambulacral discs of legs IV extend to the level of setae *h2* (Fig. 4G).

###### Remarks.

The specimens of A. (C.) limosae used here to illustrate morphological differences are those examined by Han and Min (2019a).

In contrast to the original description of A. (C.) limosae by Dubinin (1951: fig. 65) and the illustration by Vasyukova and Mironov (1991: fig. 72), the drawing of that species by Gaud (1972: fig. 26a) clearly shows that the males are characterized by an opisthosoma having distinctly S-shaped lateral margins and enlarged setae *h2*. This tends to indicate that the specimens examined by Gaud are probably those of the same species we describe herein, A. (C.) neolimosae sp. nov.

The occurrence of two closely related species of the genus *Alloptes* on *Limosalimosa* could most probably be explained by their origin from the common ancestor in different parts of the geographic range of this host. The black-tailed godwit has a very wide nesting range in Eurasia, from Iceland to Chukotka peninsular, which is split into several isolated populations in eastern part of Asia (Gill et al. 2021; Zhu et al. 2021). Since we found both mites, A. (C.) limosae and A. (C.) neolimosae sp. nov., on the same individual of *Limosalimosa*, it is possible to speculate that bird populations where these species originated are presently mixed or rejoined.

###### Etymology.

The Latin prefix *neo* (new) of the specific name reflects the close affinity to the previously described A. (C.) limosae.

###### Molecular data.

We obtained a 582 bp fragment sequence of the COI gene from four individuals of Alloptes (C.) neolimosae sp. nov. (NIBR no. NIBRIV0000895972–73, NIBRIV0000895980, NIBRIV0000895983), and the COI sequences were deposited in GenBank with NCBI accession numbers OM102971–OM102974. Intraspecific genetic distances based on 531 bp sequences of the COI gene from A. (C.) neolimosae ranged from 0.0% to 0.2%. Comparatively, interspecific genetic distances within the genus *Alloptes* ranged from 16.6% to 30.1%, with that between A. (C.) neolimosae and A. (C.) limosae being 21.7% (Table 2).

**Table 2.** Pairwise genetic distances (Kimura two-parameter) among 11 *Alloptes* species based on mitochondrial cytochrome *c* oxidase subunit I (COI) sequences.

Species (Genbank accession no.)	COI distances (%)												
	1	2	3	4	5	6	7	8	9	10	11	12	13
1. Alloptes (Conuralloptes) neolimosae sp. nov. (OM102971)													
2. Alloptes (C.) neolimosae sp. nov. (OM102972)	0.2												
3. Alloptes (C.) neolimosae sp. nov. (OM102973)	0.2	0.0											
4. Alloptes (C.) neolimosae sp. nov. (OM102974)	0.2	0.0	0.0										
5. Alloptes (Alloptes) aschizurus (MZ489638)	24.1	24.3	24.3	24.3									
6. Alloptes (Apodalloptes) orthogramme (MK456598)	20.8	21.1	21.1	21.1	26.5								
7. Alloptes (C.) calidridis (KU203101)	22.6	22.9	22.9	22.9	19.9	20.1							
8. Alloptes (C.) chionis (MZ489639)	22.5	22.8	22.8	22.8	26.4	19.9	21.0						
9. Alloptes (C.) limosae (MK456600)	21.7	21.7	21.7	21.7	25.0	20.8	20.2	21.2					
10. Alloptes (C.) procerus (MK456602)	21.0	21.2	21.2	21.2	23.5	19.7	18.7	19.2	16.6				
11. Alloptes (Sternalloptes) antarcticus (MZ489641)	24.3	24.3	24.3	24.3	25.2	22.6	21.8	26.8	24.9	24.6			
12. Alloptes (S.) fauri (MK456605)	26.0	26.3	26.3	26.3	28.6	25.9	28.2	28.4	24.9	24.6	24.6		
13. Alloptes (S.) obtusolobus (KU203100)	29.3	29.5	29.5	29.5	25.6	30.1	26.6	25.0	30.1	28.2	27.0	26.7	
14. Alloptes (S.) stercorarii (KF018833)	24.7	24.7	24.7	24.7	24.2	22.1	21.5	23.0	22.8	25.3	17.8	23.0	24.6

### ﻿Superfamily Pterolichoidea Gaud & Atyeo, 1978

**Family Syringobiidae Trouessart, 1896**

#### Genus *Phyllochaeta* Dubinin, 1951

##### ﻿ Phyllochaeta limosae sp. nov.

Taxon classificationAnimaliaSarcoptiformesSyringobiidae

EE44720D-5A1C-5CC8-AA57-B1069D909D20

http://zoobank.org/2CF088B6-94C6-4564-9CCB-EDFCCA680396

###### Type material.

**Male *holotype*** (NIBR no. NIBRIV0000895974), 1 male and 2 female paratypes (NIBR no. NIBRIV0000895975–NIBRIV0000895977) from the quills of flight feathers on wings of *Limosalimosa* (Charadriiformes, Scolopacidae), Korea, Chungcheongnam-do, Seosan-si, 37°0'12"N, 126°24'5"E, 6 July 2012, collected by Han Y.-D.

###### Description.

**Figure 5.**
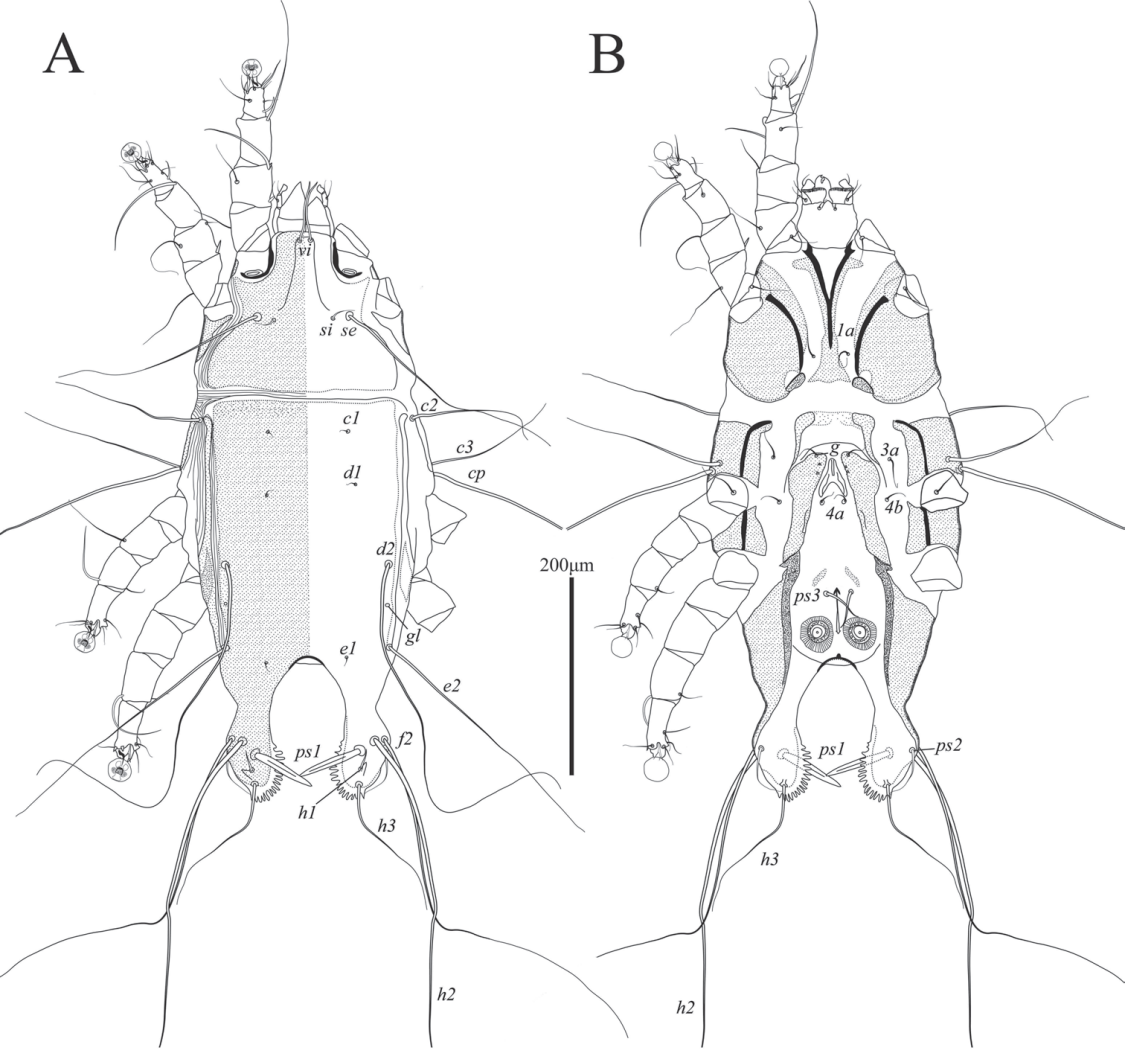
*Phyllochaetalimosae* sp. nov., male **A** dorsal view **B** ventral view.

**Male** (Figs 5, 7A–D; holotype, measurements for 1 paratype in parentheses). Length of idiosoma from anterior end to bases of setae *h3* 560 (555), greatest width 260 (245), length of hysterosoma 410 (390). Prodorsal shield: entire, with posterior margin straight; length along midline 157 (155), greatest width 195 (195), distance between setae *se* 94 (93); surface without ornamentation, anterior part with a pair of grooves flanking narrow longitudinal rectangle (Fig. 5A). Humeral shields well developed. Hysteronotal shield: anterior margin straight, length of shield from anterior end to bases of setae *h3* 390 (390), greatest width 200 (187), surface without ornamentation. Lateral sclerites fused with hysteronotal shield posterior to bases of setae *e2*. Setae *c2*, *d2*, and *e2* represented by macrosetae, 150 (160), 440 (430), and 240 (260) long, respectively. Setae *e1* situated posterior to setae *e2*, approximately at level of anterior end of terminal cleft. Opisthosomal lobes long, shovel-shaped, with distinctly enlarged distal parts, and with spine like dorsal process at bases of setae *h1*; greatest width at level of setae *f2* 50 (49). Terminal cleft large ovate, length of cleft from anterior end to bases of setae *h3* 138 (140), greatest width 81 (82), length-to-width ratio 1.7; ventral C-shaped sclerotized band at anterior end of the cleft with small median protuberance. Terminal membranes with 15 or 16 finger-like denticles along medioterminal margin. Setae *ps1* lanceolate, 69 (68) × 8 (8), situated slightly posterior to level of setae *h2*. Setae *h1* spiculiform, situated posterior to setae *ps1*. Distance between dorsal setae: *c2*:*d2* 147 (145), *d2*:*e2* 81 (83), *e2*:*h3* 142 (145), *h3*:*h3* 107 (100).

Sternum with terminal sclerotized plate shaped as a narrow triangle. Coxal fields II almost completely sclerotized, posterior tips of epimerites II with small heavily sclerotized ovate plates (Fig. 5B). Pregenital apodemes shaped as an inverted L, their anterior ends connected by poorly sclerotized transverse bridge. Paragenital sclerites large, with enlarged anterior parts, not fused anteriorly. Base of genital apparatus at midlevel of trochanters III, genital arch 39 (37) long and 30 (28) wide, aedeagus 15 (13) long. Setae *g* situated on anterior ends of paragenital apodemes, approximately at level of setae *3a* and *c3*. Setae *4a* and *4b* situated on same transverse level. Diameter of adanal suckers 24 (23), corolla with 16 or 17 round denticles. Adanal shields represented by small oblique sclerites situated anterior to setae *ps3*. Opisthoventral shields large triangular, fused anteriorly with posterior ends of paragenital sclerites. Distance between ventral setae: *4b*:*3a* 44 (40), *g*:*4b* 46 (48), *g*:*4a* 47 (50), *4a*:*ps3* 92 (85).

Setae *cG* of genua I and II spiniform. Tarsi III and IV with small apicoventral spines between bases of setae *s* and *r.* Legs IV with ambulacral discs almost extending to level opisthosomal lobe apices. Setae *d* and *e* of tarsi IV spine-like, situated on distal end of tarsus, solenidion φ of tibia IV extending to proximal margin of ambulacral disc (Fig. 7A–D).

**Figure 6.**
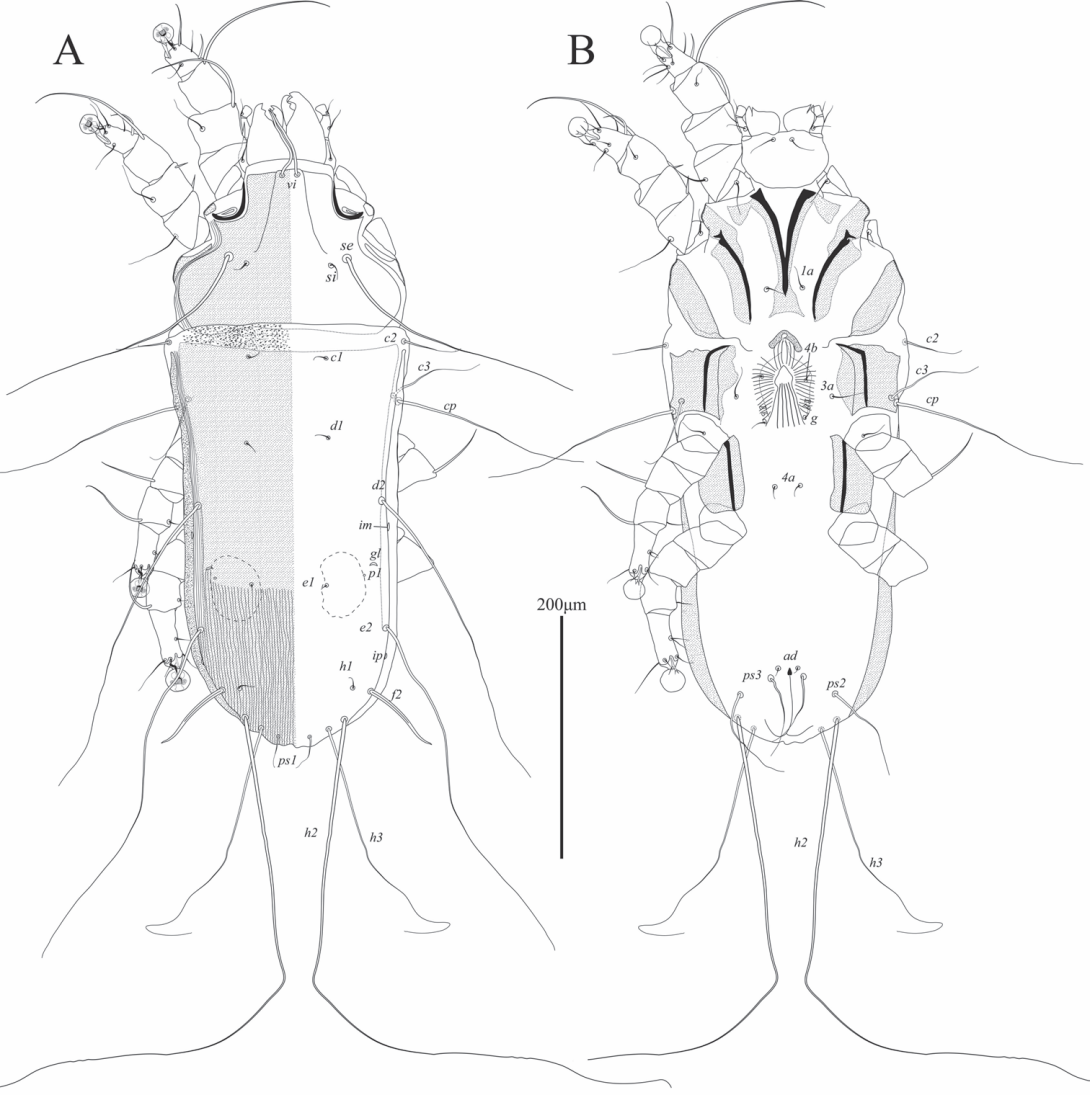
*Phyllochaetalimosae* sp. nov., female **A** dorsal view **B** ventral view.

**Figure 7.**
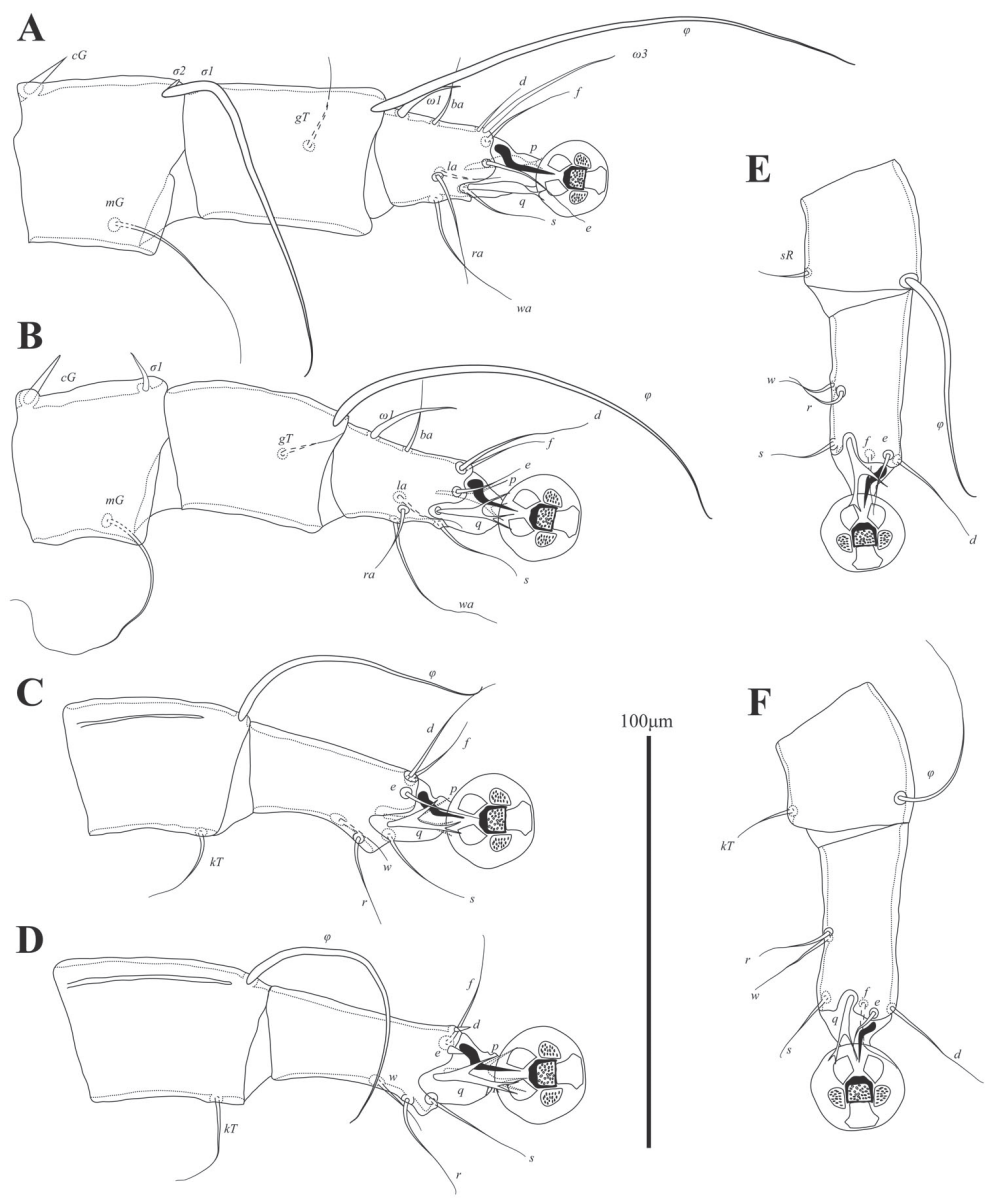
*Phyllochaetalimosae* sp. nov., legs **A** genu, tibia and tarsus I of male **B** genu, tibia and tarsus II of male **C** tibia and tarsus III of male **D** tibia and tarsus IV of male **E** tibia and tarsus III of female **F** tibia and tarsus IV of female.

**Female** (Figs 6, 7E, F; range for 2 paratypes). Length of idiosoma 475–485, greatest width 195–210, length of hysterosoma 340–345. Prodorsal shield: shaped approximately as in male, length 132–135, greatest width 182–197, distance between setae *se* 98–101; anterior part with a pair of groves flanking narrow longitudinal trapezoid (Fig. 6A). Hysteronotal shield: entire, greatest length 325–340, greatest width 180–195, surface with faint longitudinal striation in posterior one-third. Anterior ends of lateral sclerites adjacent to hysteronotal shield, posterior parts of these sclerites gradually turned onto ventral side of hysterosoma. Lateral dorsal setae *c2* short, filiform, 50–52 in length. Setae *d2* and *e2* represented by macrosetae, 280–290 and 305–320 long, respectively; setae *f2* large spiculiform, 73–76 long. Distance between dorsal setae: *c2*:*d2* 130–137, *d2*:*e2* 102–105, *e2*:*h3* 80–85, *h2*:*h2* 76–80, *h3*:*h3* 54–59.

Epimerites I and II with narrow sclerotized areas, posterior end of sternum with triangular sclerotized plate. Epigynum small, roughly semicircular, 16–18 long, 27–28 wide. Setae *g* situated posterior to level of setae *3a* and genital papillae. Setae *ad* short, 7–12 in length. Setae *ps2* and *ps3* filiform, 80–82, and 65–72 long, respectively.

Structure and setation of legs I and II as in males. Solenidion φ of leg I approximately as long as this leg. Setae *cG* of genua I and II blade-shaped. Tarsi III and IV without ventral blunt-angular extension. Solenidion φ of tibia IV slightly shorter than corresponding tarsus (Fig. 7E, F).

###### Differential diagnosis.

The genus *Phyllochaeta* currently comprises 15 species known to infest birds of the genera *Actitis*, *Arenaria*, *Calidris*, *Charadrius*, *Limnodromus*, *Prosobonia*, and *Rostratula* (Charadriiformes, Scolopacidae) (Dabert 2003). The newly described species *Phyllochaetalimosae* sp. nov., discovered here for the first time from *Limosalimosa*, and is most similar to *Phyllochaetasecunda* Dabert, 2003 described from the short-billed dowitcher, *Limnodromusgriseus* (Gmelin, 1798) (Scolopacidae), with respect to the following features: in males of both species, ornamentation of the hysteronotal shield is absent, the opisthosomal lobes are shovel-shaped with distinctly enlarged distal parts, the terminal membranes have numerous finger-like denticles along the medioterminal margin, ventral apophyses of legs I and II are absent, and genual setae *cG*I and *cG*II are spine-like; in females, dorsal setae *e1* are situated closer to the level of hysteronotal gland openings *gl* than to setae *e2*. *Phyllochaetalimosae* sp. nov. differs from *P.secunda* in the following characteristics: in males, the terminal cleft is semi-ovoid with a length-to-width ratio of 1.7, the terminal membranes have 15 or 16 finger-shaped denticles, the anterolateral extensions of the prodorsal shield are pointed, the hysteronotal shield is uniformly sclerotized, the tarsi of leg III and IV have blunt-angular ventral extensions, and setae *4a* and *4b* are approximately situated at the same transverse level; in females, the hysteronotal shield bears faint longitudinal striations in the posterior third and lacks any lacunae, and setae *c1* are situated posterior to the level of setae *c2*. In males of *P.secunda*, the terminal cleft is distinctly wider (length/width ratio about 1.0), terminal membranes have 9 or 10 finger-shaped denticles, anterolateral extensions of the prodorsal shield are widely rounded, the hysteronotal shield has a pair of longitudinal heavily sclerotized bands near the terminal cleft, ventral apophyses of tarsi III, IV are hooked, and setae *4a* are situated posterior to the level of setae *4b*; in females, the hysteronotal shield is monotonously punctate and has a pair of ovate lacunae at the level of setae *e2*, and setae *c1* and *c2* are approximately at the same transverse level.

###### Remark.

The origin of *Phyllochaetalimosae* sp. nov. on *Limosalimosa* is enigmatic and disputable. Godwits (Limosinae, *Limosa*) and curlews (Numeniinae, *Numenius*) forming most basal lineages within Scolopacidae were previously known to bear only syringobiids of the genus *Limosilichus* Vasyukova & Mironov, 1986. This genus is apparently restricted to godwits and curlews, and most its species are monoxenous (Vasyukova and Mironov 1990, 1991; Dabert 2003). While the genus *Phyllochaeta* currently including 15 species is widely distributed on waders of the tribes Arenariini, Tringini, and Scolopacini (Scolopacinae). Two species, *P.secunda* and *P.gracilis* Vasyukova & Mironov, 1986, which are close to *P.limosae* sp. nov., are specific to dowitchers *Limnodromusgriseus* and *L.scolopaceus* (Say, 1823), respectively. Therefore, it is most reasonable to hypothesize that the ancestor of *P.limosae* sp. nov. had been transferred to the black-tailed godwit from some dowitchers.

###### Etymology.

The specific name is taken from the generic name of the type host and is a noun in apposition.

## Supplementary Material

XML Treatment for Alloptes (Conuralloptes) neolimosae

XML Treatment for Phyllochaeta limosae
